# Integration of nurse-led virtual reviews with growth hormone device-linked patient adherence information: a mixed methods feasibility study

**DOI:** 10.3389/fendo.2023.1167854

**Published:** 2023-05-31

**Authors:** Helen Stokes, Julie Jones, Chris Worth, Jacqueline Nicholson, Catherine Fullwood, Indraneel Banerjee

**Affiliations:** ^1^ Department of Paediatric Endocrinology, Royal Manchester Children’s Hospital, Manchester, United Kingdom; ^2^ Department of Computer Science, University of Manchester, Manchester, United Kingdom; ^3^ Paediatric Psychosocial Service, Royal Manchester Children’s Hospital, Manchester, United Kingdom; ^4^ Research and Innovation, Manchester University National Health Service Foundation Trust, Manchester, United Kingdom; ^5^ Centre for Biostatistics, Division of Population Health, Health Services Research and Primary Care, School of Health Sciences, University of Manchester, Manchester, United Kingdom; ^6^ Faculty of Biology, Medicine and Health, University of Manchester, Manchester, United Kingdom

**Keywords:** growth hormone, growth hormone device, children, adherence, nurse-led, short stature

## Abstract

**Introduction:**

Easypod-connect™ for childhood growth disorders is a unique connected system that enables transmission of injection adherence information for recombinant human growth hormone (r-hGH). Although this system has the potential to facilitate greater adherence, observational studies have shown declining adherence over prolonged periods when used without additional support. Supplemental nurse practitioner support has been envisaged but not investigated; in this study, we have undertaken feasibility analysis of nurse-led virtual reviews (NVR) in combination with easypod-connect™ in a single centre using quantitative and qualitative analyses.

**Aims:**

We aimed to test feasibility by assessing compliance with NVR, height standard deviation score (SDS) gain, adherence improvement and patient opinions.

**Methods:**

Patients using easypod™ r-hGH were recruited prospectively to a 12-month study with two telephone NVR appointments in addition to standard of care in-person hospital outpatient visits. A subset was recruited for a semi-structured interview for qualitative thematic analysis.

**Results:**

Forty-three patients of median (range) age 10.7 (6.7, 15.2) were recruited for a period of 1.1 (0.7, 1.8) years. Thirty-three (76.7%) patients were fully compliant with NVR integration with easypod-connect™, establishing feasibility. Median (inter-quartile range, IQR) height SDS improved from -1.85 (-2.44, -1.37) to -1.48 (-2.14, -1.07) (p<0.001) while adherence remained similar in the majority from study start [96.5 (88.8, 100.0)] to end [99.0 (94.0, 100.0)]. Qualitative analysis identified themes supporting patient benefit: practicalities of appointments, perceived purpose and significance of virtual reviews, and the importance of optimising growth. Four patients complained of injection pain, of whom two switched to an alternative r-hGH device.

**Conclusion:**

Our study has demonstrated the feasibility of nurse-led virtual review integration with easypod-connect™ in a mixed methods study, laying the foundation for research in larger groups over longer periods. Nurse practitioner supported application of easypod-connect™ offers the potential for improved growth outcomes in all r-hGH devices providing adherence information.

## 1 Introduction

Recombinant human growth hormone (r-hGH) is successful therapy for growth disorders but is constrained by the requirement for regular injections over several years (1). The easypod-connect™ system has been used as a standalone and unsupported tool to identify and improve adherence with daily r-hGH injections. The system relies on transmission of injection information to a web-based platform for patient and clinician visualisation of adherence, with the intention of persuading patients to improve this. While the system does not specify action to improve adherence, the simple act of being observed is likely to increase adherence (the Hawthorne effect) (2) and this forms a key component of behaviour change techniques (3) and persuasive technologies (4, 5). In fact, the easypod-connect system directly targets several aspects of the Behaviour Change Technique Taxonomy version 1 (BCTTv1) such as 2.1 “Monitoring of behaviour by others” and 2.3 “self-monitoring of behaviour” (6). Additionally, knowledge that clinicians will review adherence allows for persuasion through the alternate routes of “Reward and Threat” (BCTTv1 10.4, 10.5, 10.10) as well as constructive feedback from clinicians to ameliorate low adherence (BCTTv1 2.2, 2.7). Importantly, the self-monitoring inherent in easypod-connect™ is well established as a persuasive tool within the healthcare setting (7, 8).

While adherence is high in the first 1-2 years using easypod-connect™, adherence levels are typically reduced beyond the first year (9, 10). This gradual reduction in the Hawthorne effect is well recognised (11) and, in the case of easypod-connect™, suggests that the system requires greater patient interaction to sustain high levels of adherence. The integration of nurse practitioner input and motivational interviewing have been suggested as supplemental tools to promote long term adherence (12, 13). It is implied, although not specified, that adherence information can be utilised in consultations by clinicians and nurses, targeting behaviour change techniques such as social support, social pressure, feedback and reward (14). However, there are no studies to evaluate clinician/nurse practitioner roles in utilising such adherence information.

Specialist nurses play a vital role in the clinical management of patients on r-hGH treatment. They have the capacity to provide support to clinicians through virtual reviews between clinic consultations and improve patient adherence to r-hGH treatment (15). While specialist nursing support to r-hGH treatment is common practice in the UK (16), supplemental nurse practitioner support to improve patient adherence and growth outcomes has not been assessed. We have proposed the logical integration of nurse-led virtual reviews (NVR) to easypod-connect™ adherence information in a prospective feasibility study. This study is intended to provide preliminary information for wider application to test adherence levels over longer periods.

## 2 Aims

The aim of the study was to prospectively test the feasibility of nurse-led virtual reviews (NVR) integrated with device-linked r-hGH adherence information. We proposed the aim to be achieved by testing compliance with NVR, measurement of growth and adherence outcomes and parent perception in qualitative analysis. An additional aim was to investigate adverse effects arising from use of the integrated easypod-connect™-NVR system.

## 3 Methods

All Royal Manchester Children’s Hospital patients with growth disorders, established for at least 4 months on r-hGH with a predicted growth period of at least 12 months (age at recruitment not exceeding 15 years) were invited to participate. All clinical indications for commencing r-hGH treatment, including those outside approved indications in the UK (17), were allowed for study participation. Informed consent was obtained as per UK research ethical approval 19/WM/02777. A control arm was absent in this feasibility study.

Sample size calculations were pragmatic, based upon a tentative number of 63 patients established on easypod™ r-hGH at the time of submission for ethical approval. Based upon a paired t-test with primary outcome height SDS, a sample size of 50 would detect a medium effect size of 0.43 with significance level 0.05 and 80% power, allowing for a 10% dropout (n=45). Calculations were undertaken in R v4.2.1 (R Core Team 2022, R Foundation for Statistical Computing, Vienna, Austria https://www.R-project.org/) using the package “pwr”;_(pwr: Basic Functions for Power Analysis, R package version 1.3-0, https://CRAN.R-project.org/package=pwr).

### 3.1 Quantitative methods

Height and weight measurements were recorded by a stadiometer and calibrated electronic weighing scales in the outpatient department of the hospital at recruitment and at the end of the study. A one-year interval was stipulated between start and end visits. In those in whom routine outpatient appointments were delayed due to prolonged waiting times (consequent to the covid-19 pandemic) or patient inconvenience, an earlier or later end appointment was allowed. In such cases, the appointment nearest to the one-year mark was taken as the end of study time point.

**Figure 1:**
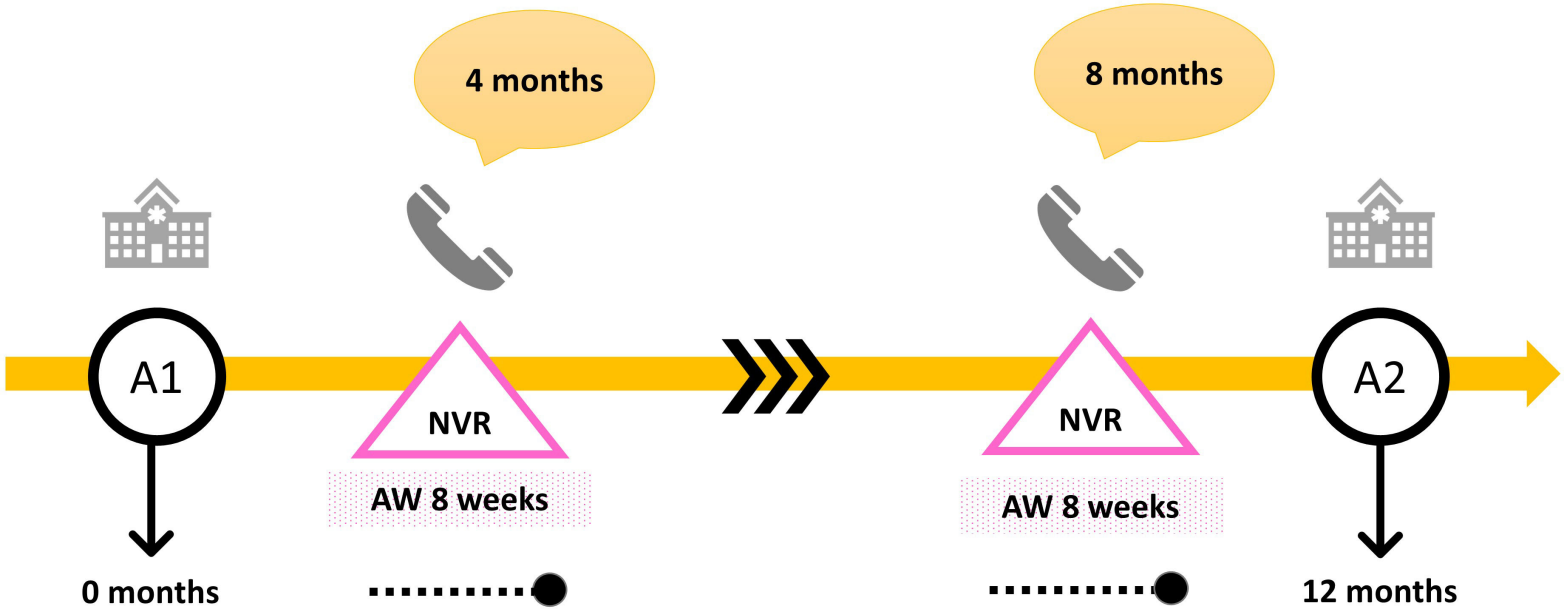
Pictorial description of study plan per patient. NVR = nurse-led virtual review conducted over a telephone call, A1 = in person appointment in hospital at the start of the study, A2 = in person appointment in hospital at the end of the study, AW = adherence window.

Two nurse-led virtual reviews (NVR) were undertaken at four and eight months (**Figure 1**) in addition to outpatient in-person appointments as per routine clinical care. The timing of NVR at four and eight months was based on an in-house retrospective audit assessing parental choice of additional NVR and practical choices based on nurse practitioner capacity, as well as timing of NVR synchronized with but not overlapping in-person outpatient visits. The frequency choice was also dependent on the recommended 2-3 times/year outpatient review standards for the treatment with r-hGH (18), corroborated with nurse practitioner suggested additional “interventions” to improve adherence (19) in between appointments and evidence of a single targeted educational “intervention” using easypod™ over a 6-month period (20). One or both parents (or those with parental responsibility) were asked to join a telephone discussion with one specialist nurse (HS or JJ). The same parent and specialist nurse participated in both NVR appointments. NVR focussed on adherence (expressed as a percentage of days when r-hGH was injected) as observed by device-linked adherence information in an adherence window in the preceding eight weeks (**Figure 1**). NVR also reviewed health and well-being, injection supplies and dose of r-hGH.

Clinical correspondence was generated from each NVR, a copy of which was sent to the parents of the child and young person by post. The NVR provided the opportunity to discuss successes and barriers to good adherence, but these were not recorded for further evaluation. At each NVR, parents were asked if they would prefer to remain in the study. If the parent(s) did not attend an NVR appointment, a further two attempts were made, following which a letter was sent to the parents to contact the research team. The frequency of NVR appointments was recorded as 0, 1 or 2, with 2 denoting full compliance with study procedure. Those with less than two NVR appointments were noted as being incompletely compliant.

Auxology measurements were converted to standard deviation scores (SDS) using UK reference ranges (21). r-hGH dose was recorded as mg/day and IGF-I levels nearest to the start and end dates were measured as mcg/L by a standard assay as per routine clinical practice. Measurement of IGF-I was not mandatory to the study protocol and IGF-I SDS was not derived due to expected range changes with age and puberty; instead IGF-I change was ascertained by paired t-tests. Statistical analysis for descriptive variables and differences between group variables was undertaken by IBM-SPSS™ version 28, using suitable parametric (t-tests and generalised linear models) and non-parametric methods (Wilcoxon, Spearman).

### 3.2 Qualitative methods

A consecutive 15 patients were approached to take part in a qualitative sub-study which involved a semi-structured interview not exceeding 30 minutes to allow parents the opportunity to share their experiences more openly. While quantitative methods tested compliance with study procedure, the qualitative arm of the study was designed to gather a richer and more in-depth understanding of parent perceptions. Interviews were conducted by two research nurses (separate from specialist nurses) over a four-week period. The research nurses were not involved in the clinical care of the patients and were therefore independent from the quantitative study protocol and performance of NVRs. Interviews took place *via* videocall (Microsoft Teams^®^) and were transcribed using the transcribing function on this platform. The transcripts were then checked for accuracy by the research nurses. The interviews followed five broad questions (**Table 1**), which had been suggested as clinically relevant by clinicians and specialist nurses in the study research team, but participants were welcome to discuss any areas they felt relevant to the subject. The interviewers checked back the understanding of participants throughout each interview.

**Table 1:** Parent interview topic guide with 5 broad questions to assist conversation in the qualitative study.

Parent Interview Topic Guide	
1. Describe in your own words your child’s participation in the study. 2. Did you find the telephone clinics useful and why? 3. Did you find anything about the telephone clinics that were not useful and why? 4. Do you have suggestions to improve the experience of telephone clinics? 5. Would you consider this method of monitoring growth hormone treatment if this becomes available to you in the future?

Thematic analysis was carried out by a clinical psychologist researcher familiar with qualitative research methods (JN), following the steps described by Braun and Clarke (22). Thematic analysis was chosen as it focuses on patterns across participants and aims to understand the participants’ lived experiences. As there is meagre existing knowledge about parental experiences of nurse practitioner reviews in the management of growth disorders, an inductive method was felt to be most appropriate. Findings were derived from the data, rather than starting with a pre-determined framework. The transcripts were read multiple times, to ensure that the researcher was fully immersed in the data and then initial codes were created. These codes were then developed into overarching themes using an interpretative process (22). The transcripts were checked a further two times to ensure that the themes captured all content and focussed on common, repeating patterns. Each theme was also checked to assess if it could be traced back to the original transcript.

### 3.3 Feasibility analysis

Study feasibility was tested by a combination of compliance with NVR, change in height SDS, change in adherence and parent perceived benefit. The study was deemed feasible if two NVR appointments (full compliance) were achieved in >50% of patients recruited to the study. Additionally, it was hypothesised that a gain in height SDS of 0.5 and a gain in adherence of 10% would support the feasibility model. In the qualitative subset of the study, it was hypothesised that the majority of patients would express themes around benefit, and this would support the conclusion of feasibility.

## 4 Results

**Figure 2:**
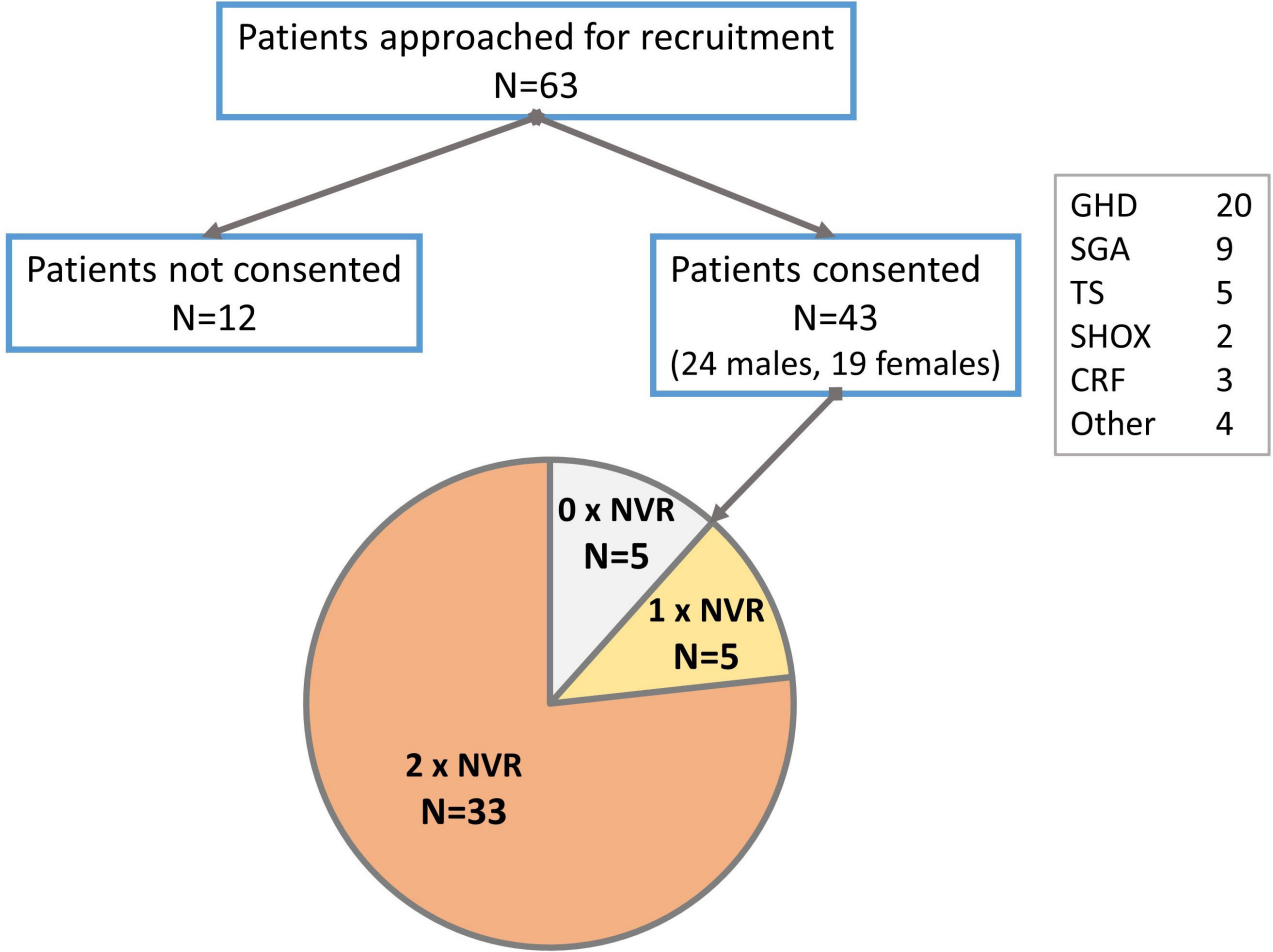
Flowchart of patients recruited to the study showing frequency of compliance with nurse-led virtual review (NVR). GHD, growth hormone deficiency; SGA, small for gestational age; TS, Turner syndrome; SHOX, short stature homeobox containing gene deficiency; CRF, chronic renal failure.

Forty-three patients (24 males, 55.8%) consented to participate in the study (**Figure 2**). Growth hormone deficiency (GHD) was the most frequent indication (46.5%) for receiving r-hGH, followed by short children born small for gestation age (SGA) (20.9%). Lesser frequencies were those with Turner syndrome (TS), short stature homeobox-containing gene deficiency (SHOX) and chronic renal failure (CRF). Four (9.3%) patients with significant short stature received r-hGH outside of UK recommendations. Thirty-three (76.7%) patients completed two NVR appointments, while five (11.6%) patients each completed either one or no NVR appointment. The median (range) ages of patients at the start and end of the study were 10.7 (6.7, 15.2) and 12.0 (7.6, 16.2) years respectively. One child recruited at age 14.9 years commenced the study at age 15.2 years and thus appeared to deviate from study protocol. At a variable point during the study period, 19 (44.2%) patients were pubertal. At the time of recruitment, patients had been on a median 28 (5, 114) months of easypod™ r-hGH treatment. The study duration was 1.11 (0.65, 1.75) years with an interquartile range (IQR) of 0.98 to 1.27 years, in keeping with the design of the study protocol.

### 4.1 Height outcomes

**Figure 3:**
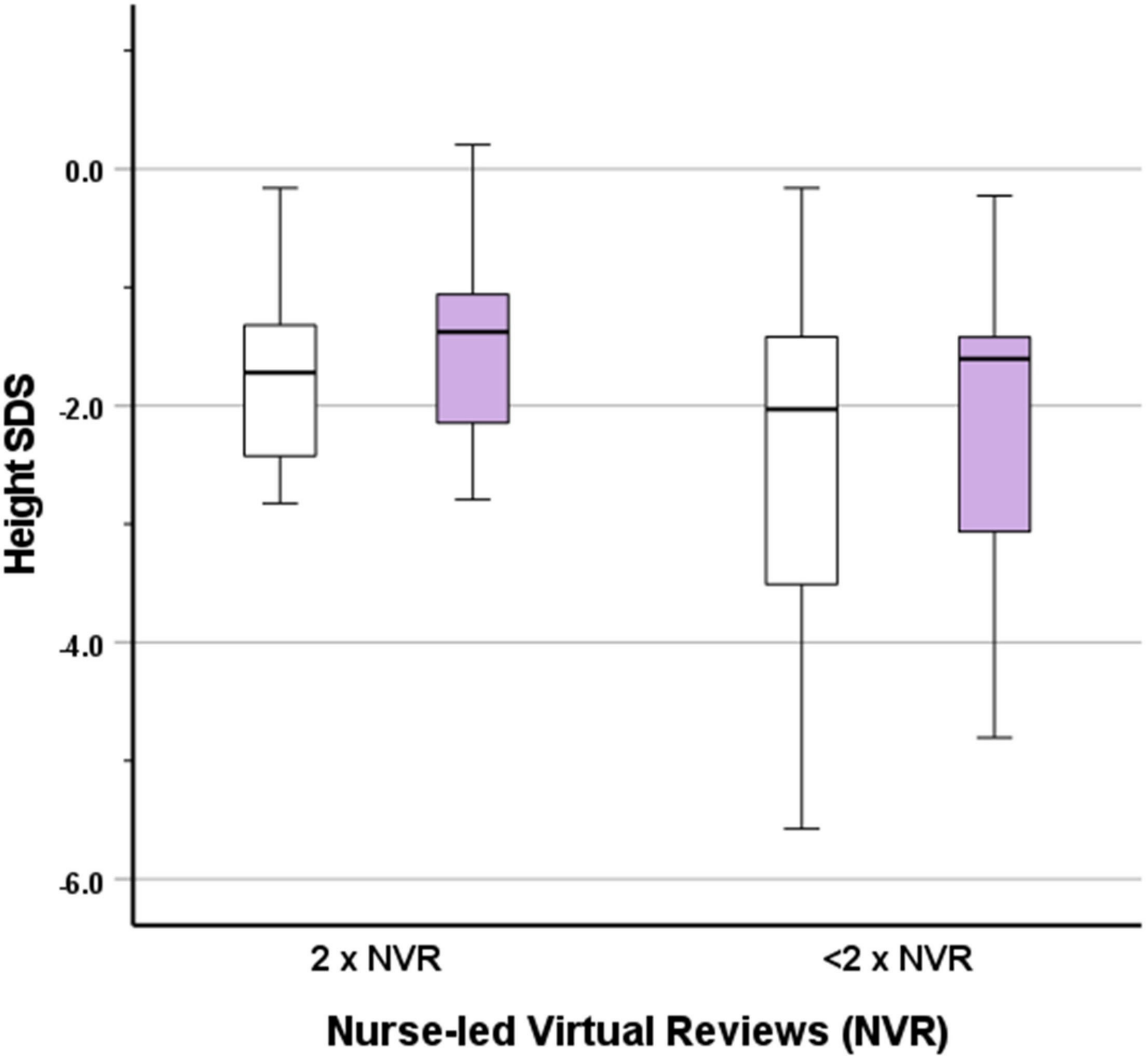
Box and whisker plot showing height SDS at the start (white boxes) and end (lilac boxes) of the study for fully compliant (2 reviews) or incompletely compliant (<2 reviews) nurse-led virtual reviews (NVR).

Recorded height and weight (and associated SDS) values at start and end of treatment are shown in **Table 2**. Height SDS improved by a median (IQR) of 0.25 (0.08, 0.40) across the whole group (**Figure 3**), indicating catch-up height gain in a large proportion of patients. The gain in height SDS showed moderate negative correlation with prior duration of r-hGH treatment (r=-0.34, p=0.029). Sub-group analysis of height SDS change in those receiving r-hGH for less than one year versus those receiving it for more than one year did not show a significant difference (0.31 [0.23, 0.45] *vs* 0.24 [0.07, 0.39], p=0.337), suggesting that a greater first year catch up height gain was not relevant to the study outcome.

**Table 2:** Median (IQR) height, weight (in recorded values and SDS) and r-hGH dose in patients recruited to the study.

	Start of study	End of study	p-value
Height (cm)	127.8 (121.0, 139.9)	137.7 (127.5, 146.0)	<0.001
Height (SDS)	-1.85 (-2.44, -1.37)	-1.48 (-2.14, -1.07)	<0.001
Weight (kg)	27.9 (22.2, 35.3)	34.2 (26.9, 43.8)	<0.001
Weight (SDS)	-1.02 (-1.57, -0.49)	-0.87 (-1.57, 0.12)	0.024
Adherence (%)	96.5 (88.8, 100.0)	99.0 (94.0,100.0)	0.282
r-hGH dose (mg/day)	0.9 (0.7, 1.2)	1.1 (0.8, 1.3)	<0.001
IGF-I (mcg/L)	248.0 (171.5, 443.0)	302.0 (200.5, 489.0)	0.112

Although adherence information was not congruent with start and end dates, the eight-week adherence window enabled correlation and convenience to record in the start and end columns. P-values are obtained using the Wilcoxon signed rank test without controlling for NVR frequency.

Adherence was measured at two nurse-led virtual review (NVR) appointments near the start and end dates by observation of device-linked adherence information.

In those fully compliant with NVR, gain in height SDS was similar to gain in the whole group. The effect of full compliance versus incomplete/no compliance with NVR on height SDS was assessed by an exploratory generalised linear model, which demonstrated non-significance (β=-0.01, p=0.911). There was no significant effect of the presence or absence of the diagnosis of GHD (β=0.09, p=0.382). Similarly, no significant effect of gender (β=0.15, p=0.138) or puberty (β=-0.05, p=0.641) was observed.

### 4.2 Adverse events

Four (9.3%) patients complained of painful injections; one (2.3%) patient switched to a different r-hGH device and therefore failed to complete the study. Another patient switched to a different r-hGH device at the end of the study. No other adverse events were noted.

### 4.3 Qualitative study outcomes

Ten (66.7%) of 15 families approached, consented to take part in a semi-structured interview with median patient age of 9.5 (7.3, 13.7) years at recruitment. Eight (80.0%) patients were female, with GHD as the predominant diagnosis (50.0%), with the remainder being small for gestational age (SGA) (30.0%) and Turner Syndrome (TS) (20.0%). Three (30.0%) patients were pubertal at or during the study period.

Each interview lasted between 10 and 20 minutes, depending on the range and extent of themes in the conversation. Three overarching themes emerged – the practicalities of appointments, the perceived purpose and significance of virtual reviews, and the importance of optimising growth. The emergence of themes and supporting quotes are provided in **Table 3**.

**Table 3:** Themes emerging from parent interview grouped into three main categories with supporting quotes in each column.

The Practicalities of Appointments	The Perceived Purpose and Significance of the Nurse-Led Virtual Review	The Importance of Optimising Growth
Difficult travelling to Manchester and back (P1) Being remote is good, daughter has a lot of appointments, more information for the caregiver, doing all the logistics and getting all of that sorted out (P2) Preference to leave the child out of the conversation. Contact does not have to be in person, less distressing for the young person, less onerous but still helpful (P2) Taking time out of school (P3) Telephone is a better option (P4) Would be interested in the future if it saved travelling to Manchester, the time it takes. Telephone is a better option, bloods could be done at the GP (P4) If its only a 5 minute phone call, every couple of months, it’s not difficult for anybody to do (P8)	Helpful having someone who knows the ins and outs of the machine Someone supervising adjusting the levels and having someone on the telephone to do that was really helpful (P2) Good to talk to someone and have contact about what’s happening with my child and for them to give advice. We spoke about diet and what we could do to help further. I was interested as hopefully this would provide more. Good to talk to someone and have someone at the other end of a phone (P3) Periodic contact with a nurse to ask a few questions and boost, make it run smoother your end (P4) Did not find phone call helpful, transferred the data, not missed a dose, that was it really, it was some time ago … not sure what would have been helpful, we know how important it is for her to have the injection every night (P4) Being able to say something rather than wait to see the specialist, good to keep in touch, just in case (P5) Submit data; how often child takes the medication. Couple of calls from the endocrine nurse. They were checking in, see how things were (P6) He’s had a few extra appointments to monitor his growth, they went through some of the settings on the device and talked through any issues (P7) It was useful in that someone was still looking at our case, rather than us just being completely left. I think as a result of it, we had a dosage change, which would only happen when we go and see the consultant (P8) It was useful to keep in contact with somebody about what’s been going on because we’ve not had a consultant visit for ages (P8) The phone calls were just to see if I had any worries or concerns (P9) One positive was that they talked about the size of the capsules, they changed them to bigger ones and the nurse got that sorted (P9) Those phone calls would also have been brilliant in the early days when child was younger and starting on growth hormone but we’ve been doing this for four years, so therefore I didn’t really have much to say (P10)	For daughter to have been measured, to have been seen locally to see the growth (P1) Maybe if she’d have had bloods done, looked at her levels, upped the dose, if it was monitored. Can we boost the growth hormone? That could have helped her to grow better (P4) It’s meant a lot to our family seeing the child grow; he’s going through a couple of sizes in shoes, he’s growing bigger, he’s putting on weight, he’s got a good appetite and he’s growing (P5) The important thing is the consultant puts up the amount of growth hormone. It seems that if the child has a growth spurt or a weight increase, we can put up the amount of the injection (P10)

De-identified patients are represented as P numbers.

#### 4.3.1 Theme 1: the practicalities of appointments

Parents were asked what they found useful about the study; they described the ease of the contact in a virtual review in contrast to the need to travel for in-person outpatient appointments in Manchester. The phone calls were of a short duration and little preparation was required, making this contact less onerous for families. Parents also spoke about the benefits of not requiring time out of school for a virtual review. However, their children were often unaware of the NVR appointment although they were aware of their enrolment into the study. Two parents suggested alternative methods for virtual reviews, such as by e-mail and text messaging.

#### 4.3.2 Theme 2: the perceived purpose and significance of the nurse-led virtual review

This was one of the main themes within the interviews with a wide variety of perspectives being shared across all ten parents. Three parents expressed that they had not found the phone calls particularly helpful. They were able to describe what the call involved but explained that it had not changed the management or routines that were already in place. This seemed to relate to different types of parents; those confident and experienced, who did not need reminders and prompts or those who felt that NVR contact was very minimal, for example, having received only one telephone call. Further, some parents were already used to having telephone contact with the specialist nurse as part of their usual care; they typically rang the nurse practitioner as and when they had any query. Some parents said they would have liked to have more contact and more information by post.

Parents who spoke very positively about their involvement in the study and the reviews highlighted specific examples of advice given or changes that were made as a result of the call. Three parents seemed to greatly value the opportunity to speak with an expert about their child’s care. Parents said “it was good to have someone at the end of a phone” and seemed to feel reassured by having “someone checking in with them” who was able to help resolve issues in a timely manner.

#### 4.3.3 Theme 3: the importance of optimising growth

A theme emerging from the majority of interviews was that the child’s growth was at the forefront of parents’ minds. Suggestions about what could have been different about the study were related to optimising the growth outcomes for their child rather than monitoring of treatment adherence. Parents talked about measurements, blood tests, monitoring and the ability to increase the medication dose in between in-person appointments with the clinician. They valued changes made at the review appointment which otherwise would have been made at infrequent in-person clinician appointments. Parents made suggestions about ways to improve monitoring; for example, heights and weights being arranged local to their residence and the information being transferred to the clinical team.

### 4.4 Outcome correlation with study aims

The study recruited 43 (86.0%) patients against a target of 50, which allowed for a dropout of 10%. Only two (4.0%) patients were missing from the primary outcome; thus, the reduction in power is negligible. With regards to the aims, the study was deemed feasible with the majority (76.7%) of parents being completely compliant with NVR. Height SDS improved over the duration of the study with a median additional height gain of 0.25 SDS (0.5 SDS expected as per hypothesis, achieved in 19.5%), although this was not attributed to independent contribution from compliance with NVR. Adherence remained high at a median level of 96.5 to 99.0% through the period of the study with no reduction noted in the majority.

In the qualitative study, ease of contact, requirement for less travel and the value of more frequent medical contact to improve growth performance in patients supported patient benefit and therefore feasibility.

Adverse events were minimal in four (9.3%) patients and involved pain during injections, requiring a switch to alternative devices in two (4.7%) patients.

## 5 Discussion

We have demonstrated the feasibility of additional nurse-led virtual reviews (NVR) using easypod-connect™ systems for the treatment of growth disorders. Easypod-connect™ has been shown to improve adherence to daily injections of r-hGH over long periods and is the only r-hGH administration system that provides device-linked adherence information. However, despite the availability of adherence information to families and caregivers with easypod-connect™, adherence levels decline progressively with prolonged use (10), suggesting that the use of self-monitoring as an isolated behaviour change technique, delivered though standalone technology, may not be sufficient to sustain adherence and optimise growth outcomes. The addition of supplemental NVR allows for the inclusion of additional behaviour change techniques such as “reward and threat” and “feedback”. The addition of more behaviour change techniques to an approach is known to improve efficacy (23) and, in this case, offers the potential to improve adherence through reinforcement of device-linked adherence information. Our feasibility study has taken the first step to integrate NVR in routine clinical care to sustain high levels of long-term adherence through multi-faceted behaviour change.

The integration of nursing support for technological advances such as telehealth is not uncommon in several specialties (24–26), accelerated partly by the covid-19 pandemic restrictions and partly by the need for better utilisation of constrained resources. The supplemental use of NVR has been suggested in recent reviews (12, 13) but a roadmap for specific use is yet to be elucidated. A nurse practitioner driven patient support programme (PSP), TUITEK^®^ was successfully trialled in Taiwan in 31 children and in 76 children in Argentina for a period of three months (27, 28); the finding of improved confidence in self-administration of easypod™ r-hGH, albeit over a short period, supports the principles of our study findings. In contrast to TUITEK^®^ findings, our study has described both quantitative and qualitative outcomes, thereby addressing perspectives of both clinicians and parents. Thus, our study results have provided the foundation for the use of a novel nurse-enhanced technology system to promote improved growth performance in patients receiving r-hGH for growth disorders.

Our study was an exploratory feasibility study designed to investigate change in height and adherence over a one-year period, but without a comparator or control arm. Thus, we were not able to definitively ascertain if additional NVR telephone calls improved height outcomes, even though an improvement in height SDS was observed between start and end dates. We will utilize the data from this feasibility study to design a future study with NVR and non-NVR groups in a cohort study to objectively assess the value of additional NVR intervention. Similarly, adherence outcomes were not increased, although the maintenance of relative high adherence in the majority suggested a positive impact. We accept that the study outcome did not meet targets as per hypothesis (gain in height SDS of 0.5 and adherence of 10%); however, a net height gain of 0.25 SD and maintenance of high adherence rates are reasonable proxies for success and do not detract from feasibility.

As the study was designed to test feasibility and did not involve a control arm, scrutiny for potential biases, for example the indications for using r-hGH, gender and puberty status, were not considered. Socio-economic status of patients was not recorded, given the nature of a feasibility study at a single centre. Further, the additional impact of other medications or the difference between self-injection and parent-administered injection was not explored, particularly in adolescent patients. These aspects will be important in future design of a larger study involving several centres with wider variation in patient preferences. Such a study will have sufficient power to test if adherence truly increases with an increasing number of NVR appointments, an important consideration for clinical translation that cannot be ascertained by analysis of the current limited dataset.

We reduced bias arising from variation in study duration by stipulating a 12-month study period, but given logistic considerations, accepted variability; nonetheless, the IQR for study duration was 1.0-1.3 years, correlating with the study protocol. We accepted the bias from duration of r-hGH treatment prior to the study; as expected there was weak negative correlation with height outcomes.

The study design introduced variability, not only in quantitative data but also in parent perceptions identified in the qualitative study. Parent responses varied in the preferred amount of contact or information from the medical team. However, themes supporting the notion of patient benefit were similar, i.e., priority to optimise growth, addressing barriers to medical management and greater convenience of access. Advice on medication dosage at NVR was particularly welcomed by parents. However, there was variation and no clear consensus in the preferred frequency of NVR appointments. Instead, as indicated above, a targeted approach was suggested with more NVR allocated to those with greater need. The study did not, however, directly address the question of whether NVR prompted greater adherence as the interview design was open and untargeted. Given our findings of the diversity of parental opinion of NVR based on self-perception of confidence, future studies may wish to assess the efficacy of NVR in those lacking intrinsic motivation and if NVR has positive independent impact. It is likely that additional behaviour change techniques and an increase in the persuasiveness of the overall, holistic approach may need to be considered.

The qualitative subset provided a parent dimension and therefore significantly enriched our quantitative study outcomes. Parent perceptions have been explored previously in patients using easypod-connect™ (29), but using a questionnaire without exploration of freely emerging themes in a qualitative design. Therefore, our study is the first to report both quantitative and qualitative arms in the use of easypod-connect™. However, our findings need to be tempered by the possibility of interviewee bias for greater NVR compliance, with all ten families completing two NVR appointments with reasonably high adherence [99 (82, 100) %] both at the start and at the end of the study [99 (75, 100) %].

No safety concerns were raised by use of NVR integration with easypod-connect™ in the full set. Four patients complained of pain during injections, prompting a switch to alternative devices in two patients. Safety concerns were not raised by parents as an emerging theme in the qualitative subset.

While the easypod-connect™ system is a significant advance in r-hGH treatment, it relies on the adoption and utilisation of digital health technology, which may be daunting to some families. In our study, we observed inadequate set up of easypod-connect™ precluding timely NVR and transmission of adherence information. It is likely that further advances in device technology, for example an automated device transmission system may facilitate a richer and more robust NVR experience.

## 6 Conclusion

We have demonstrated feasibility of the integration of nurse-led virtual review with the easypod-connect™ system for the administration of r-hGH for childhood growth disorders using quantitative and qualitative methods in a prospective, single centre study. No safety concerns were identified. This study establishes proof of principle and preliminary data for further studies integrating nurse practitioner support for emerging r-hGH adherence technology in a wider population over a longer period.

## Data availability statement

The datasets presented in this article are not readily available because Anonymised datasets may be provided upon reasonable request as per ethical approval documents. Requests to access the datasets should be directed to indi.banerjee@mft.nhs.uk.

## Ethics statement

The studies involving human participants were reviewed and approved by UK HRA 19/WM/02777. Written informed consent to participate in this study was provided by the participants’ legal guardian/next of kin.

## Author contributions

All authors contributed to the article and approved the submitted version.
